# Continuing cancer screening later in life: attitudes and intentions among older adults in England

**DOI:** 10.1093/ageing/aft132

**Published:** 2013-09-01

**Authors:** Christian von Wagner, Ana Macedo, Christine Campbell, Alice E. Simon, Jane Wardle, Victoria Hammersley, David Weller, Jo Waller

**Affiliations:** 1 Department of Epidemiology and Public Health, Health Behaviour Research Centre, University College London, Gower Street, London WC1E 6BT, UK; 2 Centre for Population Health Sciences, The University of Edinburgh, Medical Quad, Teviot Place, Edinburgh EH8 9AG, UK; 3 School of Health Sciences, City University London, College Building, Northampton Square, London EC1V 0HB, UK

**Keywords:** cancer screening stoppage, opt-in policy, older age, older people

## Abstract

**Background:** the rise in life expectancy, together with age-related increase in the incidence of most cancers, has led to mounting interest in cancer screening in older people. In England, routine invitations stop and an ‘opt-in’ (individual request) process is available from ages 71 to 76 years for breast and colorectal screening respectively. Little is known about public attitudes towards age-stoppage policy.

**Objective:** this study examined public attitudes to current stoppage policy, information preferences and intentions to request screening beyond the age of routine invitations.

**Sample:** participants (*n* = 927; age 60–74 years) were recruited as part of a TNS Research International survey and took part in home-based, computer-assisted interviews.

**Methods:** measures included: (i) attitudes towards current stoppage policy, (ii) preference for communications about screening after the end of the routine invitation period and (iii) intention to opt-in.

**Results:** the majority of respondents (78%) did not agree with age-based stoppage policies. Most (83%) wanted a strong recommendation to opt-in after this age, although the number who thought they would follow such a recommendation was much lower (27%). A majority of participants (54%) thought information on screening at older ages should come from their general practitioner (GP).

**Conclusion:** this survey indicates that older people in England wish to continue to be actively invited for cancer screening, although only a minority think that they would ultimately take up the offer. Primary care may play a role in negotiating a shared decision that is based on individual circumstances.

## Introduction

The incidence of most cancers increases with age [1, 2]. More than three in five (63%) cancers in the UK are diagnosed in people aged 65 and over, and more than a third (36%) are diagnosed in people over 75 years. Rapid increases in life expectancy [3, 4] have focused attention on cancer screening procedures and practices among older people [5–8]. In the UK, organised screening programmes are run by the National Health Service (NHS), with call–recall programmes in place for breast, cervical and colorectal (CRC) cancer. In England, routine cervical screening invitations continue to age 64 years, breast screening to 70 and CRC screening to age 75 [9]. Most international screening guidelines have similar age-based recommendations [10–12].

Screening is available beyond the age boundaries of the call–recall programmes through an ‘opt-in’ process. Within the breast screening programme, women aged over 70 are able to request mammography screening every 3 years and they are given information about how to make future appointments at their last routine screening appointment. For CRC screening, people over 75 can request a faecal occult blood test by calling the national screening telephone helpline [9]. Cervical screening is not recommended in older women who have had several consecutive previous negative tests because the incidence of disease at older ages is low and cytology can be uncomfortable for post-menopausal women; consequently, the cervical screening programme does not have an opt-in policy [13]. We, therefore, focus on breast and CRC screening in this paper.

Despite the opt-in provision, mammography screening rates are very low in women over 70 years [9], with only 4% of older women in England making use of it in 2005–2008 [14]. This could reflect a preference to discontinue screening but there is some evidence for lack of awareness of opt-in options [15, 16]. Opt-in rates in the English CRC screening pilot centres are also extremely low (<5%) [17], and no research has addressed preferences or knowledge concerning stoppage policies in the CRC screening programme. There is also little known about the support that people would like to make a screening decision after the end of routine invitations. At present, older people who contact the breast or CRC screening programme call centres are given the test they request (*J Patnick personal communication, December 2012*).

This study examined attitudes to continuing cancer screening among men and women aged 60 and over. Our objectives were to examine: (i) attitudes towards age-based stoppage policies, (ii) preference for communication about screening options after the end of the call–recall programme and (iii) intentions to opt-in to screening after the final invitation.

## Methods

Data were drawn from a population-based survey that had been commissioned and funded by the Cancer Research UK Policy Department. The survey was carried out by TNS Research International in July 2011. Random location sampling was used to select sample points across Britain, with sampling locations stratified by Government Office Region and social grade. At each location, quotas were set for age, gender and working status. Survey data were collected using home-based computer-assisted personal interviewing. Survey items were pre-tested using cognitive interviewing (*n* = 10) to ensure that they were comprehensible and clear. A complete description of the survey, including questions and response scales, is provided in Supplementary data available in *Age and Ageing* online, Appendix 1.

### Measures

#### Attitudes towards age-based stoppage policies

Respondents were asked to indicate their views about existing age-based stoppage policies by responding to a series of statements using a 5-point scale (from ‘strongly disagree’ to ‘strongly agree’). Question wording was as follows: ‘Thinking about the fact that people can carry on asking for screening once they stop receiving automatic invitations, how much do you agree or disagree with the following statements? (i) People should not have to ask for screening once they are outside the age range – they should carry on being invited automatically if screening is available to them on the NHS; (ii) I would like a strong recommendation to carry on asking for screening once I am too old to be invited automatically’.

#### Preferred source of communication

Respondents were asked to choose their preferred source of communication about cancer screening after they reach the age-limit for routine invitations. The options were: ‘(i) I would like to be told by a health professional at my last invited screening test; (ii) I would like it to be part of the general screening information leaflet; (iii) I would like a separate leaflet about it with my last screening invitation; (iv) I would like a letter from my general practitioner (GP) to tell me that I can continue to go for screening; (v) I would like to be sent information about it by Cancer Research UK’.

#### Intention to opt into screening

Respondents were asked to indicate (on a 5-point scale) how likely they were to ask for screening once they had stopped being invited automatically. This was asked as a general question and not in relation to a particular screening programme. Because of the skewed distribution, responses were coded as ‘very likely’ versus ‘all others’.

#### Self-reported previous participation in CRC and breast screening

Respondents were asked if they had accepted their last invitation from the CRC or breast screening programmes. Responses were coded as ‘yes’ or ‘no/don't know’.

#### Socio-demographic variables

Respondents were asked to indicate their gender and age. Social grade was indexed with the National Readership Survey social grade classification system based on occupation (or previous occupation if receiving an occupation/private pension), grouped into AB (managerial/professional); C1 (supervisory); C2 (skilled manual) and DE (semi-skilled/unskilled manual and state pensioners or casual/lowest grade workers or unemployed). People who were not working were classified according to the chief wage earner in the household.

### Analysis

The sample was divided into 5-year age bands (60–64; 65–69; 70–74 years). After descriptive analyses, we used multivariate logistic regression models to examine predictors of attitudes and intention to opt-in. To examine the role of previous breast screening participation, we ran separate analyses (adjusting for all demographic characteristics) in the female respondents. For each model, we calculated adjusted odds ratios (OR) and 95% confidence intervals (CIs). We used Chi-square tests to examine associations between socio-demographic factors and preference for the source of communication. Analyses were carried out using SPSS 19.0 (IBM, Chicago, IL, USA). Data were weighted to ensure that demographic profiles matched those for adults in Great Britain aged 50 or over in terms of gender, age, region, social grade and working status.

## Results

### Sample characteristics

Responses from 927 men and women aged 60–74 years living in England were included in the analysis (mean age = 66.3, SD = 4.4). Just over half the respondents were women (55%) and they were distributed across all social grade groups (AB: 31%; C1: 24%; C2: 22%; DE: 23%). Most respondents were married/living as married (67%).

Overall, 54% (502/927) of respondents (men: 57%; women: 52%) said they accepted their last invitation for CRC screening. Among women, 85% (*n* = 430/506) said they responded to their last invitation for breast screening.

### Attitudes towards age-based stoppage for all screening programmes

As shown in Table 1, the majority of respondents (78%) agreed that people should not have to ask for screening once they are outside the age range, and 83% wanted a strong recommendation from the NHS to opt into screening after this age. Demographic variables were not significant predictors of agreement with these statements but previous screening participation was (see Table 1). Men and women who had responded to their most recent invitation for CRC screening were more likely to want a recommendation to opt-in than those who had not responded (88 versus 76%; adjusted OR: 2.17, 95% CI: 1.51–3.10, *P* < 0.001). In a separate multivariate logistic regression analysis including only women (adjusting for age, social grade and marital status), those who had responded to their last breast screening invitation were more likely to want a recommendation (85 versus 65%; adjusted OR: 2.96, 95% CI: 1.65–5.32, *P* < 0.001).

**Table 1.** Predictors of attitudes towards age-based stoppage

	(i) People should not have to ask for screening once they are outside the age			(ii) I would like a strong recommendation to carry on asking for screening		
	Univariate analysis		Multivariate analysis	Univariate analysis		Multivariate analysis
	Unadjusted % strongly agree/agree	Unadjusted OR (95% CI)	Adjusted OR (95% CI)	Unadjusted % strongly agree/agree	Unadjusted OR (95% CI)	Adjusted OR (95% CI)
All (*n* = 927)	78.1			82.6		
Age						
60–64 years (*n* = 385)	78.7	Reference	Reference	83.1	Reference	Reference
65–69 years (*n* = 286)	79.2	1.03 (0.71–1.51)	1.03 (0.71–1.51)	86.3	1.27 (0.82–1.96)	1.23 (0.79–1.91)
70–74 years (*n* = 256)	76.0	0.87 (0.59–1.27)	0.92 (0.62–1.35)	77.5	0.70 (0.47–1.04)	0.73 (0.49–1.11)
Gender						
Male (*n* = 419)	80.0	Reference	Reference	82.9	Reference	Reference
Female (*n* = 507)	76.5	0.81 (0.59–1.12)	0.86 (0.62–1.19)	82.3	0.95 (0.67–1.34)	1.01 (0.70–1.44)
Social grade						
AB (*n* = 286)	80.3	Reference	Reference	85.2	Reference	Reference
C1 (*n* = 225)	77.5	0.85 (0.55–1.30)	0.90 (0.59–1.39)	82.7	0.83 (0.52–1.34)	0.90 (0.55–1.46)
C2 (*n* = 204)	77.0	0.83 (0.54–1.29)	0.88 (0.56–1.37)	79.7	0.69 (0.43–1.11)	0.76 (0.47–1.24)
DE (*n* = 212)	77.2	0.83 (0.54–1.29)	0.93 (0.59–1.46)	81.8	0.79 (0.49–1.28)	0.91 (0.55–1.50)
Marital status						
Married/living as married (*n* = 621)	80.1	Reference	Reference	84.0	Reference	Reference
Not married/living as married (*n* = 306)	73.9	0.70 (0.51–0.97)*	0.76 (0.54–1.06)	79.5	0.73 (0.51–1.05)	0.86 (0.59–1.24)
CRC screening participation						
Yes (*n* = 502)	80.3	Reference	Reference	87.9	Reference	Reference
No (*n* = 425)	75.4	0.76 (0.55–1.04)	0.80 (0.58–1.11)	76.0	0.44 (0.31–0.62)**	0.46 (0.32–0.66)**

Adjusted OR, odds ratios adjusted for all demographic variables; CI: confidence interval.

*Significant at *P* < 0.05.

** Significant at *P* < 0.001.

### Preferred source of communication about screening

Most respondents (54%) would prefer a letter from their GP explaining that they could continue to have screening once invitations stopped (see Table 2). This preference was slightly stronger among those from lower SES groups (DE: 58%; C2: 63%; C1: 50%; AB: 47%; *χ*^2^ (3) = 13.97, *P* = 0.003). Preference for source of communication about screening did not vary by age group, gender or marital status.

**Table 2.** Distribution of preferences for a source of communication about screening after the end of the programme (*n* = 927)

	*n* (%)
I would like a letter from my GP to tell me that I can continue to go for screening	499 (53.8)
I would like to be told by a health professional at my last invited screening test	124 (13.4)
I would like it to be part of the general screening information leaflet	101 (10.9)
I would like a separate leaflet with my last screening invitation	99 (10.7)
I would like to be sent information about it by Cancer Research UK	44 (4.8)
Don't know	60 (6.5)

### Intention to opt into screening after the end of the programme

After being given the information that opt-in was available, respondents were asked how likely they would be to ask for screening once they had stopped being invited automatically. Overall, 27% of respondents said they would be ‘very likely’ to request screening, 33% said they would be ‘quite likely’ and 13, 12 and 10% were ‘neither likely or unlikely’, ‘quite unlikely’ and ‘very unlikely’ to request screening respectively. Age, gender, social grade and marital status were not significantly associated with a strong intention (being ‘very likely’) to request screening (see Table 3).

**Table 3.** Predictors of intention to opt into screening

	Logistic regression model for predicting strong intention to opt-in		
	Univariate analysis		Multivariate analysis
	Unadjusted %	Unadjusted OR (95% CI)	Adjusted OR (95% CI)
All (*n* = 927)	26.8		
Age			
60–64 years (*n* = 385)	26.3	Reference	Reference
65–69 years (*n* = 286)	30.1	1.21 (0.86–1.69)	1.16 (0.82–1.64)
70–74 years (*n* = 256)	23.9	0.88 (0.61–1.27)	0.92 (0.63–1.34)
Gender			
Male (*n* = 419)	25.0	Reference	Reference
Female (*n* = 507)	28.4	1.19 (0.89–1.60)	1.29 (0.95–1.75)
Social grade			
AB (*n* = 286)	29.7	Reference	Reference
C1 (*n* = 225)	26.3	0.86 (0.58–1.27)	0.92 (0.62–1.38)
C2 (*n* = 204)	27.0	0.88 (0.59–1.31)	0.98 (0.65–1.47)
DE (*n* = 212)	23.6	0.73 (0.49–1.10)	0.82 (0.54–1.26)
Marital status			
Married/living as married (*n* = 621)	27.9	Reference	Reference
Not married/living as married (*n* = 306)	24.8	0.86 (0.63–1.17)	0.97 (0.70–1.35)
CRC screening participation			
Yes (*n* = 502)	34.9	Reference	Reference
No (*n* = 425)	17.2	0.39 (0.29–0.53)*	0.40 (0.29–0.55)*

Adjusted OR, odds ratios adjusted for all demographic variables; CI: confidence interval.

*Significant at *P* < 0.001.

Respondents who had participated in their most recent CRC screening round were more likely to intend to opt into screening than those who had not (35 versus 17%; adjusted OR: 2.51, 95% CI: 1.83–3.45, *P* < 0.001). In a separate multivariate logistic regression analysis, women who had responded to their most recent breast screening invitation were also more likely to intend to opt-in (32 versus 9%; adjusted OR: 4.10, 95% CI: 1.84–9.11, *P* = 0.001).

## Discussion

This study explored attitudes towards age-based stoppage screening policies, information preferences and intentions to request screening after the end of the routine invitations among older adults in England. Both men and women were surveyed, extending previous research that has largely focused on mammography.

Most respondents held positive attitudes towards continuing screening after the end of the call–recall programmes; consistent with a previous study showing that the majority of British women aged over 70 would like to continue receiving automatic invitations for mammography screening after the end of the routine programme regardless of health status [15]. These findings are in line with evidence of high general public enthusiasm for cancer screening [18].

Interestingly however, only just over a quarter of respondents (27%) strongly intended to carry on with screening. This contrasts with a US study in which most adults (72%) aged 70 or more reported that they planned to continue CRC screening throughout their lives [19]. Nevertheless the number of people intending to carry on with screening in our sample is still considerably greater than the current opt-in rates in England. It is possible that individuals change their minds once they get older, but perhaps more likely that they forget to arrange screening or forget how to request it when they stop being invited automatically. However, it is also plausible that as the cohort surveyed here reaches the age of routine screening invitation stoppage, they may want to know more about opt-in opportunities. Indeed, a third of respondents reported that they would be ‘quite likely’ to carry on with screening. With increasing emphasis on improving information and communication about screening, national programmes may need to review the information they supply on opt-in policies.

It is also possible that the relatively low levels of intention to continue screening may in part reflect some level of understanding that the benefits of screening at older ages are likely to be moderated by health status and life expectancy [20]. An individual's ability to tolerate the required treatment, and implications for future quality of life, complicate the decision-making process [6]. The decision whether or not to continue screening for a specific cancer may require support from health professionals in primary care to enable a more personalised or ‘negotiated’ recommendation [21, 22]. We know that the UK public value a recommendation from the NHS to attend screening [23], but GPs may be called on to have more direct involvement in screening decisions in older adults. As the views of the GP can influence patient choices, either explicitly through endorsement [24], or through more subtle mechanisms [25–27], there is a need for guidance for GPs and other healthcare providers on discussing screening options with their patients who are reaching the upper age-limit of screening programmes [28, 29].

### Strengths and limitations

The population sampling frame and large sample size made it possible to examine demographic as well as attitudinal predictors in this under-investigated area. In common with other surveys, respondents may have been more positively disposed towards screening than the general population; however, the presentation of this survey made it clear it was covering a range of issues, so specific attitudes to screening were unlikely to have biased participation. Confidence in the sample representativeness is increased by the finding that self-reported CRC screening participation among eligible respondents (54%) was in line with uptake rates in England [30], although mammography screening rates were higher than the national coverage figure of 73% [9]. In terms of interpreting people's responses, they were not provided with information on any age-specific advantages and disadvantages of screening, which may have limited their ability to make informed responses. Finally, while this survey focused on the potential contribution of primary care, other healthcare professionals especially geriatricians may also play an important role in facilitating decisions about cancer screening and treatment in later life.

## Conclusion

This study indicates a preference among older people in England to continue to be invited for cancer screening beyond the current stoppage age. Although the proportion that expressed an intention to take up the invitation (27%) was comparatively low, it was a great deal higher than current observed uptake rates (<4%). In the future, health professionals may be asked to play a role in supporting individual screening decision-making taking account of age- and health-specific evidence on harms and benefits.

**Key points**

- With increasing life expectancy, cancer screening practices in older adults are attracting greater interest.
- Little is known about public attitudes towards age boundaries and opt-in opportunities.
- This survey identified a clear preference among older adults in England to continue to be actively invited for cancer screening.
- Only one in four respondents (27%) said they would be very likely to carry on with screening beyond the stoppage age.
- The majority of participants wanted guidance from their GP.

## Conflicts of interest

None declared.

## Funding

This study was funded by the Cancer Research UK Policy Department.

## Supplementary data

Supplementary data mentioned in the text is available to subscribers in *Age and Ageing* online.
